# ABCA1 haplodeficiency affects the brain transcriptome following traumatic brain injury in mice expressing human APOE isoforms

**DOI:** 10.1186/s40478-018-0569-2

**Published:** 2018-07-26

**Authors:** Emilie L. Castranio, Cody M. Wolfe, Kyong Nyon Nam, Florent Letronne, Nicholas F. Fitz, Iliya Lefterov, Radosveta Koldamova

**Affiliations:** Department of Environmental and Occupational Health, University of Pittsburgh, Pittsburgh, PA 15261 USA

**Keywords:** Traumatic brain injury, Apolipoprotein E, ABCA1, Transcriptome, WGCNA, Microglia sensome

## Abstract

Expression of human Apolipoprotein E (*APOE*) modulates the inflammatory response in an isoform specific manner, with *APOE4* isoform eliciting a stronger pro-inflammatory response, suggesting a possible mechanism for worse outcome following traumatic brain injury (TBI). APOE lipidation and stability is modulated by ATP-binding cassette transporter A1 (ABCA1), a transmembrane protein that transports lipids and cholesterol onto APOE. We examined the impact *of Abca1* deficiency and APOE isoform expression on the response to TBI using 3-months-old, human *APOE3*^*+/*+^ (*E3/Abca1*^+/+^) and *APOE4*^+/+^ (*E4/Abca1*^*+/+*^) targeted replacement mice, and *APOE3*^*+*/+^ and *APOE4*^+/+^ mice with only one functional copy of the *Abca1* gene (*E3/Abca1*^*+/−*^; *E4/Abca1*^+/−^). TBI-treated mice received a craniotomy followed by a controlled cortical impact (CCI) brain injury in the left hemisphere; sham-treated mice received the same surgical procedure without the impact. We performed RNA-seq using samples from cortices and hippocampi followed by genome-wide differential gene expression analysis. We found that TBI significantly impacted unique transcripts within each group, however, the proportion of unique transcripts was highest in *E4/Abca1*^+/−^ mice. Additionally, we found that *Abca1* haplodeficiency increased the expression of microglia sensome genes among only *APOE4* injured mice, a response not seen in injured *APOE3* mice, nor in either group of sham-treated mice. To identify gene networks, or modules, correlated to TBI, *APOE* isoform and *Abca1* haplodeficiency, we used weighted gene co-expression network analysis (WGCNA). The module that positively correlated to TBI groups was associated with immune response and featured hub genes that were microglia-specific, including *Trem2, Tyrobp, Cd68* and *Hexb.* The modules positively correlated with *APOE4* isoform and negatively to *Abca1* haplodeficient mice represented “protein translation” and “oxidation-reduction process”, respectively. Our results reveal *E4/Abca1*^*+/−*^ TBI mice have a distinct response to injury, and unique gene networks are associated with *APOE* isoform, *Abca1* insufficiency and injury.

## Introduction

Traumatic brain injury (TBI) is a significant public health concern; it is a major cause of death and disability in the United States, and its occurrence is highest among multiple vulnerable populations, including the elderly, young adults, and military personnel [16]. No treatment currently exists for the approximately 2 million cases of TBI sustained each year in the United States, and the costs of medical care for 2010 were estimated at $76.5 billion annually [10].

TBI is caused by an initial external force, whether a physical object or inertia, contacting the head [27]. The impact and initial mechanical stress placed on the cells constitute the primary injury, whereas the secondary injury occurs after the inciting traumatic event, and involves multiple pathways and signaling cascades that can cause further damage [3, 13, 39]. Inflammation is a major component of the secondary injury. Inflammation is present in every case of TBI and may be a driving force for secondary pathology [13]. Chronic neuroinflammation following TBI was closely associated with neuronal death and impaired cell proliferation in locations both immediately adjacent to, and more distant from, the site of injury [1]. Many studies have shown that levels of inflammation and inflammatory molecules are strongly correlated with multiple measures of outcome in patients, including neurobehavioral impairments and survival rates [29, 45]. Microglia are the brain’s main form of immune response to infection, disease, and injury, as well as the source of inflammation. As such, inflammation and microglia have been recent concentrations of research as a means of developing therapies and improving outcomes of TBI.

Outcomes of TBI include possible changes in cognition, behavior, emotion, and sensory processing, all of which are influenced by injury severity and location [5, 18, 35]. Additionally, research has linked TBI to the risk of developing neurodegenerative diseases, including chronic traumatic encephalopathy and Alzheimer’s disease (AD) [36]. The high level of heterogeneity in outcomes suggests a significant role for genetic influence on brain susceptibility and recovery [15, 44]. The apolipoprotein E (*APOE*) gene has been frequently studied to determine its role in TBI and its isoform-dependent impact on outcome. The *APOEε4* allele is the strongest genetic risk factor for late onset AD, and is thought to confer worse outcome after TBI [2]. *APOEε4* carriers have been found to have slower recovery, increased risk of posttraumatic seizures, and worse memory performance after TBI [2, 12, 14]. However, multiple studies also show that *APOEε4* carriers did not differ from non-carriers in cognitive performance, functional outcomes or recovery after TBI [11, 37]. The contradictory results so far emphasize the need for more research on APOE and TBI.

APOE is involved in several pathways after a TBI occurs, including inflammation [20]. Inheritance of the *APOEε4* allele is associated with increased inflammatory responses, including after TBI [28]. APOE4 may induce a more robust pro-inflammatory reaction from microglia and may suppress anti-inflammatory signaling [4, 23, 24, 26]. This may be a result of decreased stability and faster catabolic degradation of APOE4*,* compared to the other isoforms, which is possibly due to its lower lipidation levels [20]. APOE is secreted as nonlipidated apolipoprotein, cholesterol and phospholipid efflux to lipid-poor APOE is mediated by ATP Binding Cassette Transporter A1 (ABCA1) [7]. *Abca1* deficiency results in decreased APOE lipidation and APOE levels [17, 21]. ABCA1 may also play a role in modulating the inflammatory response in the brain. Mice lacking brain ABCA1 saw increased inflammatory gene expression, and the microglia cultured from these mice exhibited an increased pro-inflammatory response, as seen by higher levels of TNFα secretion and lower phagocytic activity, in response to lipopolysaccharide administration [19]. It is not known how *Abca1* haploinsufficiency may influence TBI.

We recently performed transcriptional profiling of APOE expressing mice after TBI using Next Generation Sequencing [9]. Using a network-based approach, we were able to identify distinct modules correlated to injury and APOE isoform, as well as a module driven by APOE isoform across TBI groups. The aim of this study was to examine the effect of *Abca1* haploinsufficiency on gene expression induced by TBI in APOE targeted replacement mice using transcriptional profiling and a network-based approach. We used 3-month-old mice expressing human *APOE3*^*+/+*^ and *APOE4*^*+/+*^ isoforms (*E3/Abca1*^+/+^ and *E4/Abca1*^+/+^, respectively), and compared them to their *Abca1* haploinsufficient counterparts (*E3/Abca1*^+/−^ and *E4/Abca1*^+/−^, respectively), after performing a controlled cortical impact. Transcriptional profiling of hippocampal and cortical tissue from the injury site was performed using RNA-sequencing (RNA-seq). *E4/Abca1*^*+/−*^ mice had higher expression levels of the common up-regulated transcripts after TBI, which included genes related to the immune response and inflammatory response. We then examined how ABCA1 insufficiency impacted expression of the microglia sensome genes, and found that *E4/Abca1*^*+/−*^ TBI mice expressed these genes higher than *E4/Abca1*^*+/+*^ TBI mice, whereas no difference was found when comparing sham *Abca1*^+/−^ to *Abca1*^+/+^ mice of either isoform. There was no effect of *Abca1* haploinsufficiency on the expression of microglia genes in *APOE3* TBI mice. We were able to correlate the transcriptome to each phenotype using a network-based approach, Weighted Gene Co-expression Network Analysis (WGCNA). We found that the immune response module, although correlated positively to all TBI groups regardless of APOE isoform or *Abca1* copy number, consisted of genes expressed at higher levels in *E4/Abca1*^*+/−*^TBI mice, and featured microglia-specific hub genes, including *Trem2, Tyrobp, Hexb,* and *Cd68.* Our results demonstrate an effect of ABCA1 deficiency on microglia gene expression after TBI in *APOE4* mice.

## Materials and methods

### Animals

All animal experiments were approved through the University of Pittsburgh Institutional Animal Care and Use Committee and carried out in accordance with PHS policies on the use of animals in research. Human *APOE3*^*+*/+^ and *APOE4*^+/+^ targeted replacement mice (referred to as *E3/Abca1*^+/+^ and *E4/Abca1*^+/+^) were bred to *Abca1*^*+/−*^ mice to generate *APOE3*^+/+^/*Abca1*^+/−^ and *APOE4*^+/+^/ *Abca1*^*+/−*^ (referred to *E3/Abca1*^+/−^ and *E4/Abca1*^+/−^, respectively) [8, 17]. All mice were on the C57BL/6 genetic background and experimental groups consisted of both genders. Experimental mice were kept on a 12 h light-dark cycle with ad libitum access to food and water. At 3 months of age, these mice were randomly assigned to either sham or controlled cortical impact (CCI) experimental group. Mice were handled for 2 days (5 min per day) prior to surgical procedures. All materials were purchased through ThermoFisher Scientific, unless otherwise noted.

### Traumatic brain injury

CCI model of brain injury was performed as previously described [9]. Anesthesia was induced using 5% isoflurane, after which it was maintained at 1.5% isoflurane. The head was secured using a stereotaxic frame, and core body temperature was held at 37 °C using a heating pad. After shaving the heads, two separate iodine - alcohol washes were performed to sterilize the surgical site. A 50% mixture of bupivacaine and lidocaine was applied to the area and ophthalmic ointment was applied to the eyes. The scalp was opened with a midline incision exposing the dorsal aspect of the skull and the skull leveled. A 4.5 mm diameter craniotomy was performed over the left parietal cortex using a dental drill. Once the bone flap was removed, mice in the CCI group received a single impact at 1.0 mm depth with a 3.0 mm diameter metal tip onto the cortex (3 m/s, 100 ms dwell time; Impact One, Leica). Sham mice received identical anesthesia and craniotomy, but did not receive impact and are considered negative controls. Following the impact, the surgical site was sutured, triple antibiotic cream applied, Buprenex (0.1 mg/kg, IP) provided for analgesia, and sterile saline administered for rehydration. Mice were allowed to recover on heating pad, until freely mobile, before returning to their home cage.

### Tissue processing

Fourteen days post-injury, mice were anesthetized using Avertin (250 mg/kg of body weight, i.p.) and perfused transcardially with 20 mL of cold 0.1 M PBS pH 7.4 [9, 31]. Brains were rapidly removed and a 1.5 mm coronal section of the brain, including the injury site, taken by slicing the brain at − 2.5 mm and − 4.0 mm from bregma. Within the coronal slice, the hemispheres were separated, and the subcortical tissue was dissected out; hippocampal and cortical tissue were snap-frozen together for RNA isolation and RNA-seq.

### RNA isolation and RNA sequencing

All procedures were performed as before [9, 32]. CCI and sham mice consisting of both genders for each genotype were used for RNA-seq. RNA was isolated from frozen cortices and hippocampi at the injury site and purified using RNeasy kit (Qiagen) according to the manufacturer recommendations. Quality control of all RNA samples was performed on a 2100 Bioanalyzer instrument (Agilent Technologies) and samples with RIN > 8 were further used for library construction using RNA Library Prep Reagent Set (Illumina). Libraries for Next Generation Sequencing were generated by PCR enrichment including incorporation of barcodes to enable multiplexing. Sequencing was performed by the Next Generation Sequencing Center (University of Pennsylvania, https://ngsc.med.upenn.edu/) on HiSeq 2500 machine. Following initial processing and quality control, the sequencing datasets were further analyzed for differential gene expression, which in all cases was calculated using Subread/featureCounts (v1.5.0; https://sourceforge.net/projects/subread/files/subread-1.5.0/) for read alignment and summarization and statistical package edgeR (v3.14.0; https://bioconductor.org/packages/release/bioc/html/edgeR.html). Lists of differentially expressed genes are further analyzed as described in the following section.

### Weighted gene co-expression network analysis (WGCNA)

Network analysis was performed using WGCNA (v.1.51; https://cran.r-project.org/web/packages/WGCNA/index.html) [33, 48]. Libraries are clustered by gene expression enabling the detection of outliers and the power is determined by scale free topology model. Modules were generated automatically using a soft thresholding power, β = 10, a minimum module size of 18 genes and a minimum module merge cut height of 0.25. Modules were named by conventional color scheme and then correlated with trait data using Pearson’s correlation (APOE isoform, Injury, *Abca1* copy number). Statistical significance was determined by student’s *t*-test, *p* < 0.05. All modules were summarized by module eigengenes (ME), the first principle component of each module that was calculated as a synthetic gene representing the expression profile of all genes within a given module.

Representative networks were built using hub genes and the transcripts connected to them. Hub genes were identified using cutoffs of their interconnectivity within the module (module membership, > 0.8), and the correlation between expression level and trait (gene significance, > 0.2). Once the hub genes are selected, the connections to other transcripts are sorted by weight, with the first 150 connections used for visualization. Gene-association networks of interest were visualized using Cytoscape (v3.3.0). Unsupervised hierarchical clustering was performed on ME turquoise using pheatmap (v1.0.10; https://cran.r-project.org/web/packages/pheatmap/index.html) to identify 2 sub-modules. A representative network was built for each sub-module consisting of only genes within the sub-module.

### Functional pathway analysis

Functional annotation clustering was performed using the Database for Annotation, Visualization and Integrated Discovery (DAVID v6.8, https://david.ncifcrf.gov) [25].

## Results

### TBI induces changes to the transcriptome that are common among both *Abca1*^*+/+*^ and *Abca1*^*+/−*^ mice expressing human APOE isoforms

**Fig. 1:**
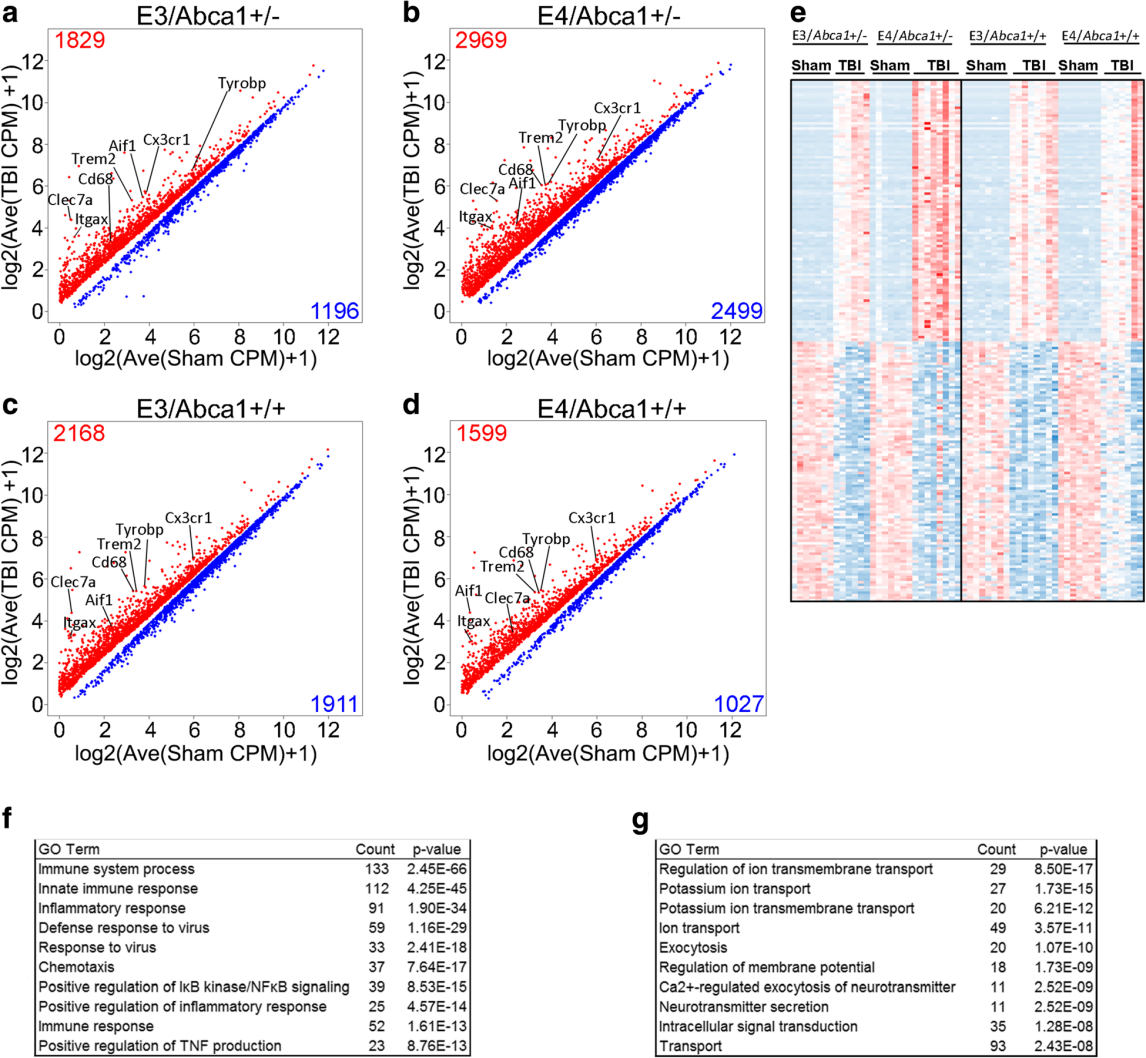
TBI increases the expression of genes associated with immune response, and decreases the expression of genes connected to ion transmembrane transport. RNA was isolated from the hippocampal and cortical tissues collected 14 days after injury from *Abca1*^*+/−*^ and *Abca1*^*+/+*^ mice of both *APOE* isoforms and was then used to perform RNA-seq, *N* = 6–8 mice per group of both genders. **a**-**d** Scatter plots represent the RNA-seq results for differentially expressed genes. EdgeR analysis between sham and injured mice identified significant affected transcripts in (**a**) *APOE3/Abca1*^+/−^, (**b**) *APOE4/Abca1*^+/−^, (**c**) *APOE3/Abca1*^+/+^ and (**d**) *APOE4/Abca1*^+/+^ mice. Red denotes up-regulated, and blue denotes down-regulated genes, *p* < 0.05. **e** Heatmap of the top 100 upregulated and downregulated genes by TBI is shown. **f** A table shows the top annotated GO terms derived from the common, upregulated genes (total = 1215 genes). **g** A table shows the top annotated GO terms derived from the common, downregulated genes (total = 531 genes)

To examine the effect of TBI on gene expression in the brains of *Abca1*^*+/−*^ and *Abca1*^*+/+*^ mice expressing human APOE isoforms (*E3/Abca1*^*+/−*^*, E4/Abca1*^*+/−*^*, E3/Abca1*^*+/−*^*, E4/Abca1*^*+/+*^), we collected hippocampal and cortical tissue from the injury site at 14 days post-injury. Total RNA was isolated from these tissues and used for RNA-sequencing. As shown in Fig. 1a-d, TBI significantly affected the transcriptome within each genotype. We highlighted several genes of interest on the scatterplots increased by TBI, and while they were differentially expressed within all the groups, the group with the highest CPM values was the *E4/Abca1*^*+/−*^ TBI mice. To determine what similarities existed among the affected biological processes, we examined the differentially expressed genes that were significant and common among the 4 genotypes. The expression levels of the top 100 up- and down-regulated genes are shown in the heatmap (Fig. 1e). Although the genes are common, the *E4/Abca1*^*+/−*^ mice show the highest expression levels of the upregulated genes. Figure 1f shows the biological processes associated with the common, upregulated genes. There were 1196 up-regulated genes common to all the groups and the top Gene Ontology (GO) terms derived from these genes were “immune system process”, “innate immune response”, and “inflammatory response”. In comparison, Fig. 1g shows the biological processes associated with the common, downregulated genes. There were 579 downregulated genes common to the groups, and these genes were functionally associated with “regulation of ion transmembrane transport”, and “potassium ion transport”.

### TBI significantly alters the expression of transcripts unique to each genotype, with the highest proportion of unique transcripts among *E4/Abca1*^*+/−*^ mice

**Fig. 2:**
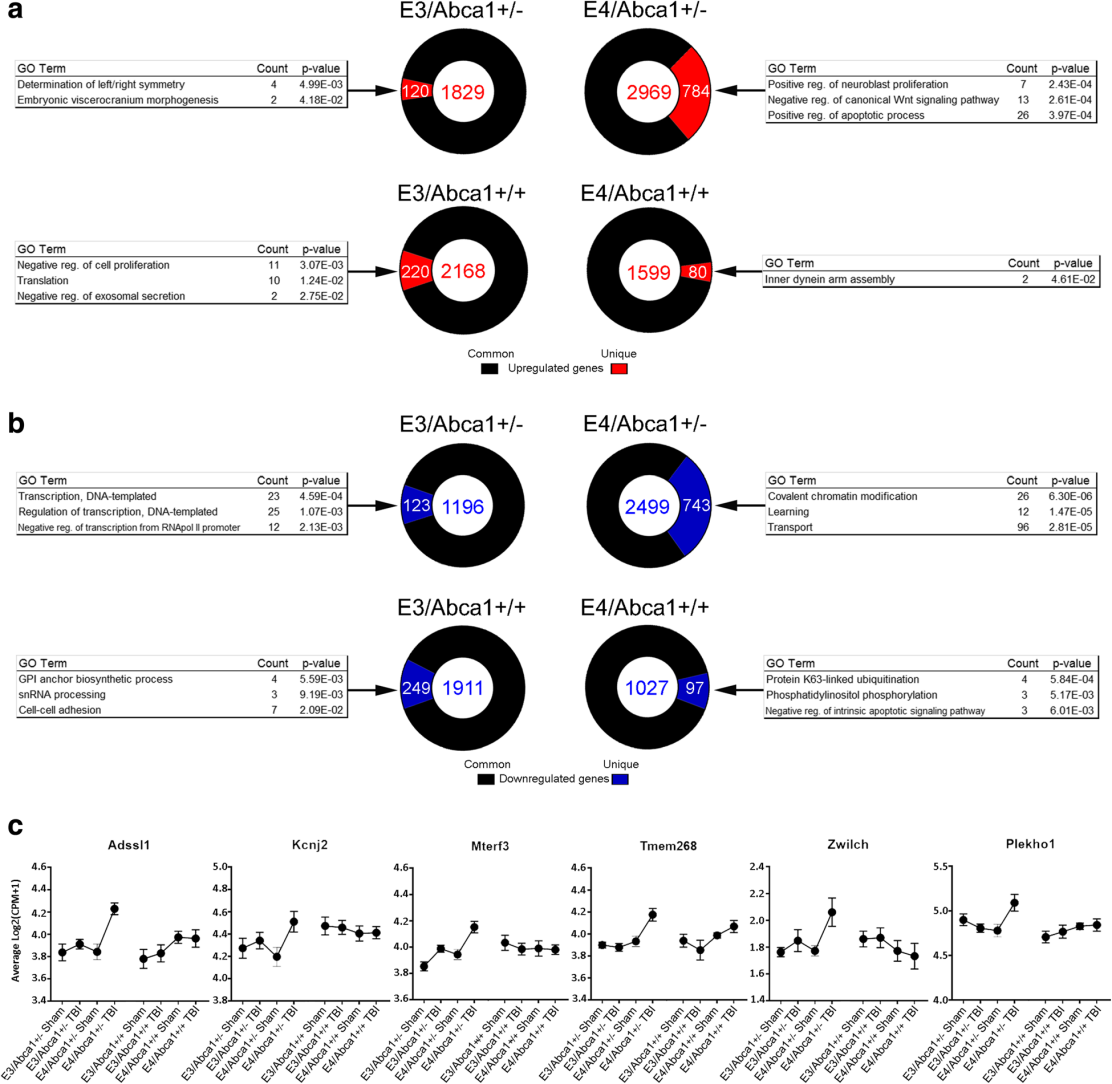
TBI affects a greater proportion of unique genes in *E4/Abca1*^*+/−*^ mice. **a**, **b** Donut plots for each genotype indicate the proportion of significantly (**a**) up- or (**b**) down-regulated genes that are either expressed in common among 2 or more groups (black), or unique to that group (**a**: red and **b**: blue). The total number of genes are shown within the center of the plot for each genotype. The top 3 GO terms for the unique genes in each genotype are shown to either side. **c** Expression plots for unique transcripts upregulated in *E4/Abca1*^*+/−*^ mice are shown

We were interested in whether the response to TBI was specifically influenced by each genotype, particularly by *Abca1* haplodeficiency in conjunction with *APOE4* isoform. To do this, we determined the proportion of genes that were differentially expressed, either in common among several of the groups or were uniquely expressed in only one group. These proportions are shown for each genotype in the donut plots in Fig. 2. As seen in Fig. 2a, *E4/Abca1*^*+/−*^ mice have a higher proportion of unique transcripts (26%) that are up-regulated by TBI than the other genotypes (*E3/Abca1*^+/−^: 5.5%; *E3/Abca1*^+/+^: 10%; *E4/Abca1*^*+/+*^: 5.0%). The biological processes derived from the unique genes of the *E4/Abca1*^+/−^ mice, include “positive regulation of neuroblast differentiation” and “positive regulation of apoptotic process”. The biological functions associated with the unique, upregulated genes within each group differ greatly; the other top terms include “determination of left/right symmetry”, “negative regulation of cell proliferation”, and “inner dynein arm assembly” for *E3/Abca1*^+/−^, *E3/Abca1*^+/+^, *E4/Abca1*^+/+^respectively. Expression plots show the distinct upregulation in *E4/Abca1*^+/−^ TBI mice of several genes, including *Plekho1*, which has been shown to promote apoptosis (Fig. 2c) [47].

*E4/Abca1*^+/−^ mice, again, have a higher proportion of unique, significant down-regulated transcripts (30%) than the other genotypes (*E3/Abca1*^+/−^: 10%; *E3/Abca1*^+/+^: 13%; *E4/Abca1*^+/+^: 9.4%) (Fig. 2b). The top GO terms derived from the unique down-regulated genes for each group are “transcription, DNA-templated”, “covalent chromatin modification”, “GPI anchor biosynthetic process”, and “Protein K63-linked ubiquitination” for *E3/Abca1*^+/−^, *E4/Abca1*^+/−^, *E3/Abca1*^+/+^, *E4/Abca1*^+/+^, respectively.

### *Abca1* haploinsufficiency upregulates microglia sensome genes in injured *APOE4* mice

**Fig. 3:**
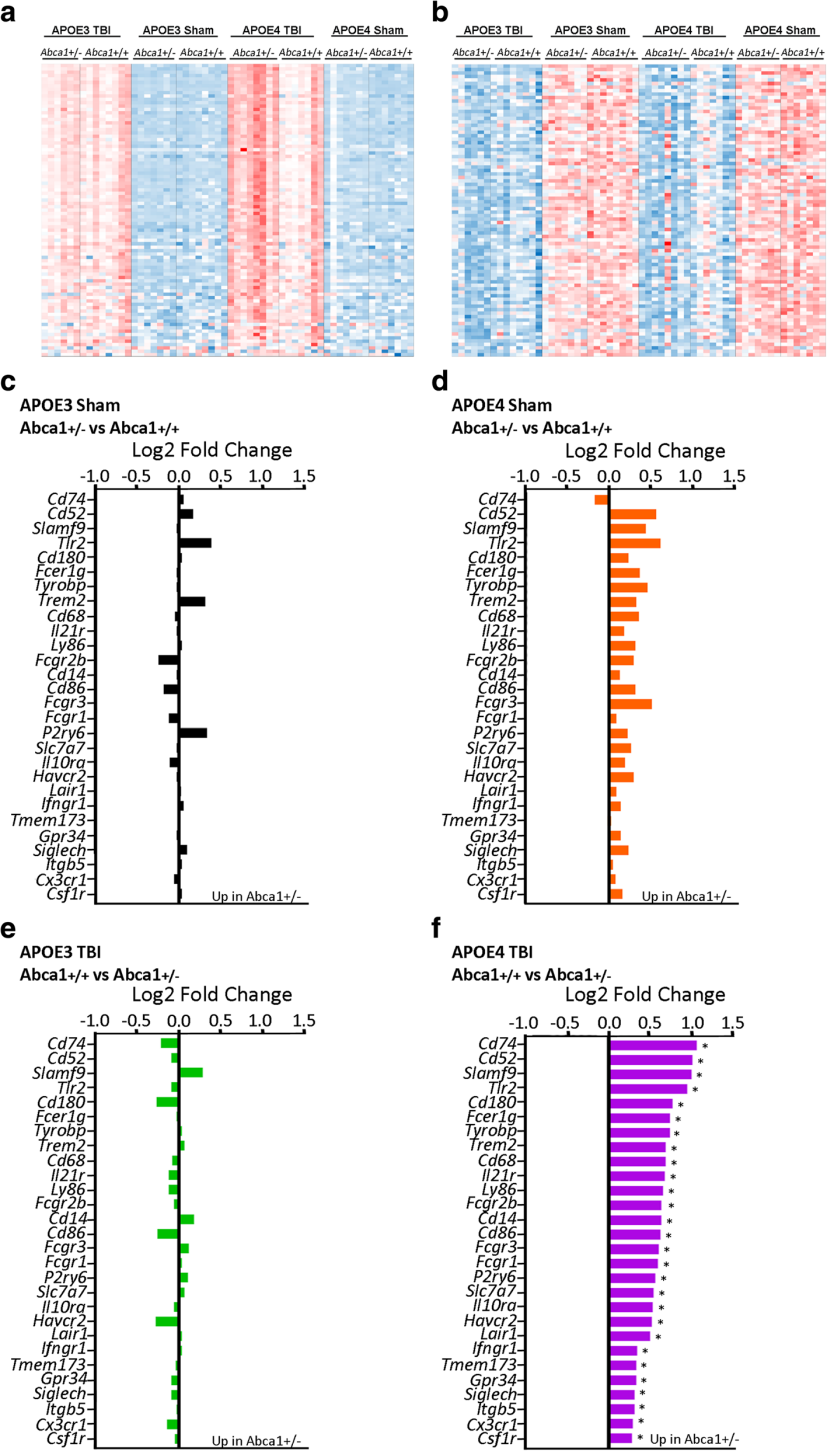
*Abca1* deficiency affects the microglial response to TBI in an *APOE* isoform-dependent manner. **a**-**b** Heatmaps were generated using normalized *Abca1*^*+/−*^ versus *Abca1*^*+/+*^ CPM values for each group for (**a**) microglia sensome genes and (**b**) synaptic transmission genes. Red denotes higher expression values, and blue denotes lower expression values. *n* = 6–8 per group, including both males and females. **c**, **e** Selected genes from the microglia sensome of *APOE3/Abca1*^+/−^ and *APOE3/Abca1*^+/+^ mice are compared separately for (**c**) sham (black bars) and (**e**) TBI groups (green bars). Shown are the Log2-fold change values for each gene. **d**, **f** Selected genes from the microglia sensome of *APOE4/Abca1*^+/+^ and *APOE4/Abca1*^+/−^ mice are compared separately for (**d**) sham (orange bars) and TBI groups (purple bars). Shown are the Log2-fold change values. *: *p* < 0.05

To determine if there was any effect of *Abca1* haploinsufficiency on gene expression changes induced by injury, we examined the expression of microglial sensome genes. Although a clear effect of TBI is present in the differential expression of the microglia sensome by *Abca1* genotype, the heatmap also shows that *E4/Abca1*^+/−^ TBI mice have higher expression levels of microglial sensome genes than the other groups (Fig. 3a). In contrast, there is no effect of *Abca1* copy number on synaptic transmission genes (Fig. 3b), although an injury effect on expression is still visible. We examined the expression levels of the microglia sensome genes within each *APOE* isoform, separated by injury status, for the effect of *Abca1* genotype. Sham mice in both *APOE* isoforms (Fig. 3c-d) and injured *APOE3* mice (Fig. 3e) have no significant changes in microglia sensome gene expression due to *Abca1* haploinsufficiency. In comparison, the injured *E4/Abca1*^*+/−*^ mice demonstrate significant expression of the microglia sensome compared to *E4/Abca1*^*+/+*^ TBI mice (Fig. 3f). In conclusion, these results demonstrate an effect of *Abca1* haploinsufficiency on the microglia sensome in *APOE4* mice after TBI.

### WGCNA reveals interconnected gene clusters associated with each trait of interest- *APOE* isoform, *Abca1* copy number, and injury status

**Fig. 4:**
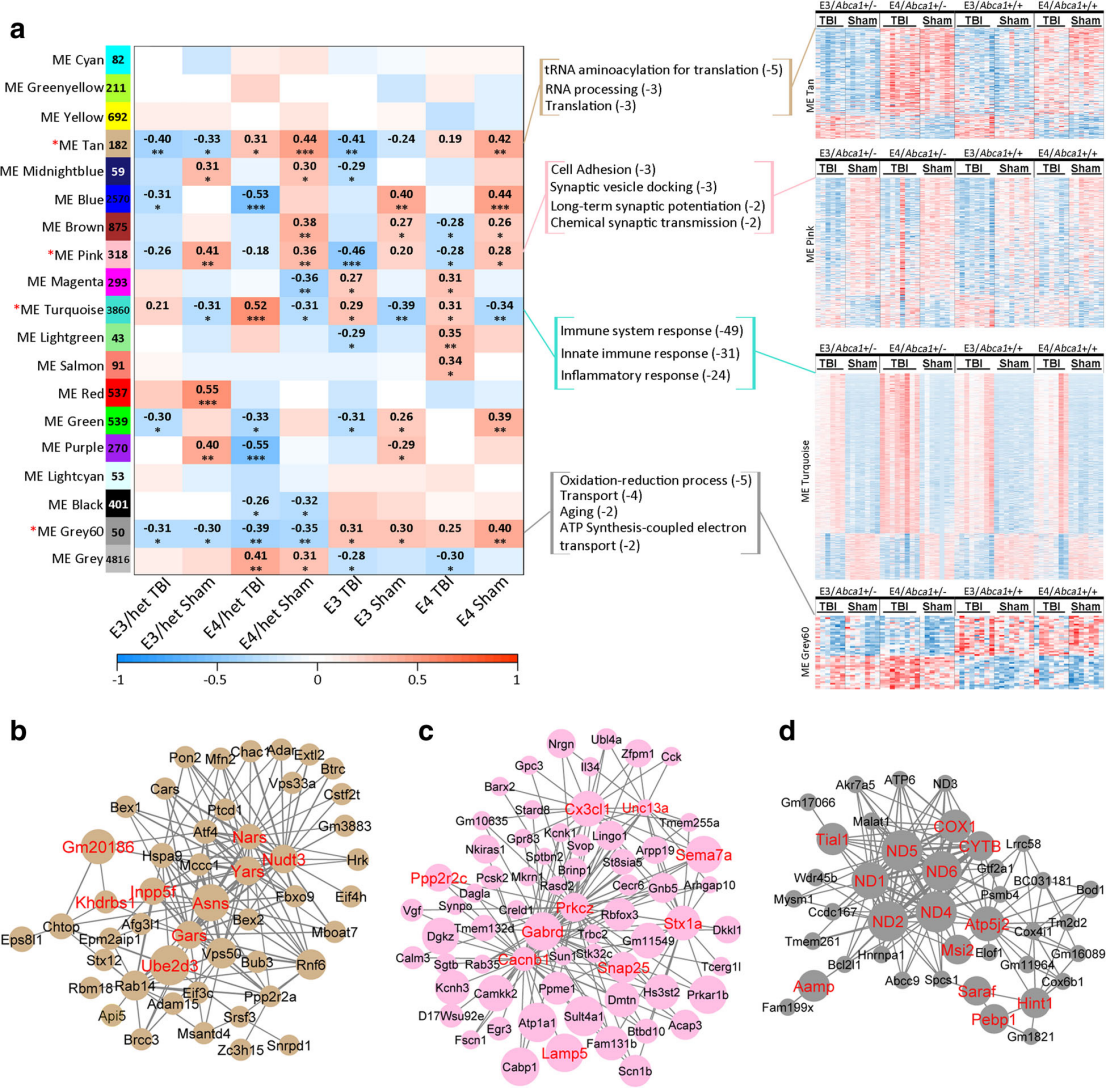
WGCNA identified modules correlated to TBI and *Abca1* haploinsufficiency. WGCNA was used to determine the correlation of module eigengenes to Injury and *Abca1* genotype. **a** The relationship tables shows the correlation between the module eigengene (rows) and group (columns) with *p*-value. Red denotes a positive, and blue is a negative correlation. Modules of interest are differentially expressed between trait conditions. MEs turquoise and pink correlated with TBI in opposite directions, ME tan correlated with *APOE* isoform, and ME grey60 with *Abca1* genotype. Top assigned GO terms and their log10 of the *p*-values are shown to the right of the table, aside heatmaps of the genes within each module, for each animal. Red denotes higher expression values, and blue denotes lower expression values. **b**-**d** Representative networks for (**b**) ME tan, (**c**) ME pink, and (**d**) ME grey60 were built using module hub genes. Hub genes are identified in red font. Size of the nodes represents the module membership value and the width of the edge, the interaction between genes shown as connecting lines, represents the weight of the connection

To identify interconnected gene clusters, or modules, associated within each trait of interest, we employed WGCNA. We were interested in the modules that were differentially expressed across our traits of interest - injury status, *APOE* isoform and *Abca1* copy number. The relationship table (Fig. 4a) shows the MEs of interest and the corresponding correlation coefficients per group.

ME tan (module size = 182 genes) correlated across the groups depending on *APOE* isoform, regardless of either injury status, or *Abca1* copy number. It positively correlated to *APOE4* groups and negatively with *APOE3* groups. As seen in the module heatmap (Fig. 4a; far right), the gene members are generally increased in APOE4 mice and decreased in APOE3 mice. The GO terms associated with the module genes were “tRNA aminoacylation for translation”, “RNA processing”, and “translation”. We built a representative network using the hub genes associated with “tRNA aminoacylation for translation”, such as *Yars, Gars,* and *Nars,* which are aminoacyl-tRNA synthetases.

ME pink correlated with injury status, however, it negatively correlated to TBI groups and positively correlated with sham groups. The biological processes associated with ME pink (module size = 518 genes) included “synaptic vesicle docking”, “long-term synaptic potentiation”, and “chemical synaptic transmission”, which suggests that injury decreases synaptic transmission. The representative network (Fig. 4c) was built around several hub genes related with synaptic transmission, including *Stx1a, Snap25,* and *Lamp5*, which are all associated with synaptic vesicle docking and neurotransmitter release. *Lamp5,* in particular, is associated with GABAergic synaptic transmission and short-term synaptic plasticity [40]. Another hub gene is *Prkcz,* which is necessary for long-term potentiation in hippocampal CA1 pyramidal cells [42].

ME grey60 correlated with *Abca1* copy number, specifically, it negatively correlated with *Abca1*^+/−^ mice and positively correlated with *Abca1*^+/+^ mice, regardless of injury or *APOE* isoform. As seen in Fig. 4d, the network was built around hub genes, which represented GO terms “oxidation-reduction process”, “transport”, and “aging”. These hub genes included a number of the NADH hydrogenase subunits, such as *ND1*, *ND2*, *ND4*, *ND5* and *ND6*. Other hub genes were *COX1, Atp5j2,* and *CYTB*. All of these hub genes are involved in the mitochondrial respiratory chain [46].

**Fig. 5:**
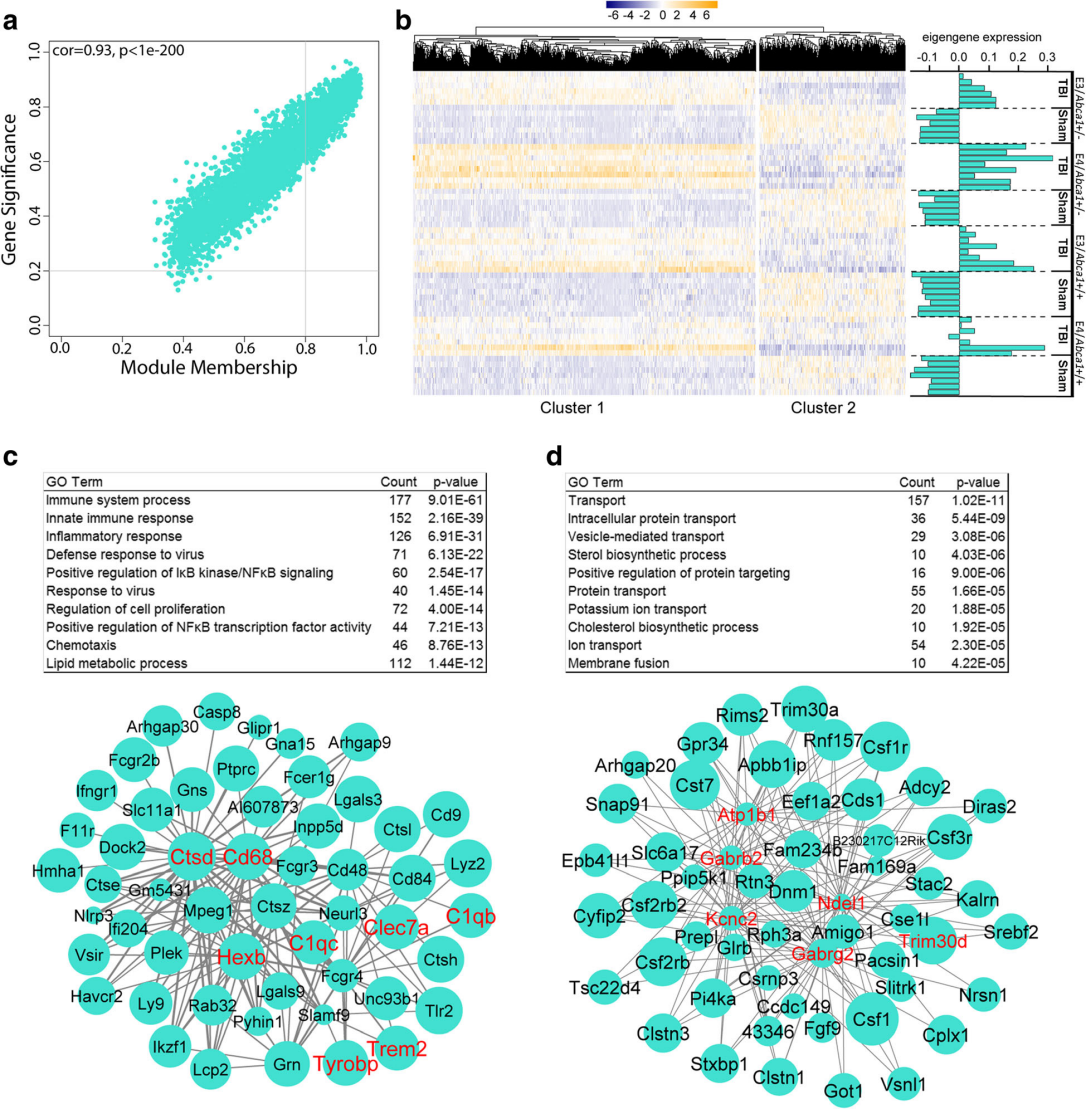
ME turquoise consists of two sub-modules correlated to injury status. **a** Scatter plot for ME turquoise module showing the correlation between module membership and gene significance. The degree of association between MM and GS was evaluated by Pearson correlation. Correlation value and p value are indicated in the plot. **b** The pheatmap shows normalized gene expression values beside module eigengene expression values for each sample for ME turquoise. The pheatmap function aggregated the module into 2 sub-modules (Cluster 1 and Cluster 2) by hierarchical clustering. **c**-**d** Tables of top assigned GO terms are shown above representative networks for (**c**) cluster 1 and (**d**) cluster 2. Hub genes are identified in red font. Size of the nodes represents the module membership value and width of the edge represents the weight of the connection

ME turquoise strongly correlated with injury status, however, unlike ME pink, it correlated positively to TBI groups and negatively to sham groups. The genes within ME turquoise are strongly connected and related to the module biological process, as seen in the module membership and gene significance scatterplot (Fig. 5a). Due to the size (module size = 3860 genes), we were interested in further separating the module. To do this, we ran a pheatmap function on the genes within the module, which aggregates the genes using hierarchical clustering. As shown in Fig. 5b, the pheatmap separated the module into 2 distinct clusters based on injury status and direction of expression. Additionally, the pheatmap shows the expression for all the genes in ME turquoise and the eigengene expression for each sample. The first cluster, Cluster 1, (size = 2605 genes) consisted of genes upregulated in TBI groups and downregulated in sham groups. The pheatmap suggests a stronger response of the cluster 1 genes within the *E4/Abca1*^*+/−*^ mice, which is consistent with the correlation of ME turquoise to this group in the relationship table. The GO terms derived from Cluster 1 were “immune system response”, “innate immune response”, and “inflammatory response”. Additionally, among the top 10 GO terms was “lipid metabolic process”. The representative network (Fig. 5c) was built around hub genes associated with immune response, such as *Clec7a, C1qc,* and microglia-specific genes, *Trem2, Tyrobp, Hexb,* and *Cd68.*

The second cluster, Cluster 2, (size = 1111 genes) featured genes downregulated in the TBI mice, upregulated in the sham mice. Functionally, this cluster is enriched in genes connected to the GO term “transport”, other transport-associated terms, such as “vesicle-mediated transport”, but also GO terms “sterol biosynthetic process” and “cholesterol biosynthetic process”. The network (Fig. 5d) built for Cluster 2 excluded any genes from Cluster 1, and functionally represents transport, however, while hub genes, *Gabrb2, Gabrg2,* and *Atp1b1* all relate directly to the transport of ions across the membrane, through this mechanism, these genes are also strongly associated with synaptic transmission. In conclusion, ME turquoise strongly correlated to injury status, but hierarchical clustering of the genes revealed two distinct clusters associated with the gene expression direction. Cluster 1 was larger and featured genes related to immune response and was more strongly upregulated in *E4/Abca1*^*+/−*^ mice, while cluster 2 featured genes downregulated in TBI groups and represented transport, but functionally are also involved in synaptic transmission.

## Discussion

We examined the impact of *Abca1* deficiency and *APOE* isoform expression on the response to traumatic brain injury. Our goal was to identify differences in the transcriptional response and trait-associated genome-wide correlated gene networks between *Abca1*^*+/+*^ and *Abca1*^*+/−*^ mice following a controlled cortical impact in human *APOE3*^*+/+*^and *APOE4*^*+/+*^ targeted replacement mice. We found that the four groups within our study -*E3/Abca1*^+/−^, *E4/Abca1*^+/−^, *E3/Abca1*^+/+^, and *E4/Abca1*^+/+^ − had common and distinct responses to TBI. *E4/Abca1*^+/−^ mice had the highest proportion of unique transcripts affected by TBI, suggesting that *E4/Abca1*^+/−^ mice are more disposed to changes in gene expression by TBI than the other groups, and demonstrate possible pathways that could be associated with worsened outcome, such as downregulated genes associated with learning. The common, up-regulated genes were associated with biological processes related to immune response, innate immune response and inflammatory response. While, these genes were common among the four groups, the *E4/Abca1*^+/−^ mice had higher expression levels of the genes upregulated by TBI compared to the other groups, suggesting a role for *APOE* isoform and ABCA1 in the expression of inflammatory genes after TBI. Consequently, we examined the effect of *Abca1* insufficiency on microglia sensome genes by injury status and APOE isoform. When comparing injured *Abca1*^+/−^ to *Abca1*^+/+^ mice, we found *E4/Abca1*^+/−^ TBI mice had increased expression of the microglia sensome genes. In contrast, there was no effect of *Abca1* copy number in *APOE3* mice, sham or TBI. These results suggest that *Abca1* haploinsufficiency may influence the inflammatory response following TBI, particularly through an impact on microglia and their gene expression. This effect is seen only among *APOE4* mice, not *APOE3* mice; this response may be related to the isoform-specific effect on inflammation. Additionally, the *APOE4* isoform may be more vulnerable to the consequences of *Abca1* haploinsufficiency due to a gene-gene interaction, a result also demonstrated by data from AD-model mice [17]. These results suggest a possible mechanism for worse outcome after TBI associated with *APOE4* isoform.

Using WGCNA, we identified modules associated with each trait – injury, *APOE* isoform and *Abca1* copy number. ME tan was associated with *APOE* isoform; the module positively correlated with *APOE4* mice and negatively correlated with *APOE3* mice, regardless of *Abca1* copy number or injury status. The representative network was associated with the GO term “tRNA aminoacylation for translation”, and included hub genes *Yars, Gars,* and *Nars,* which are aminoacyl-tRNA synthetases. Mutations in these genes are associated with Charcot-Marie-Tooth disease, one of the most commonly inherited neurological disorders [6]. Additionally, a metabolomics study on AD patient CSF and plasma found that a pathway significantly affected in plasma by AD severity was aminoacyl-tRNA biosynthesis, however, the mechanisms associated with altered aminoacyl-tRNA synthetases and AD remain unknown [41].

The “synaptic transmission” module, ME pink was significantly correlated and down-regulated by injury across the groups. The network represented GO terms “synaptic vesicle docking”, “long-term synaptic potentiation”, and “chemical synaptic transmission”. The hub genes featured in the representative network, included *Stx1a, Snap25,* and *Lamp5*, which are all associated with synaptic vesicle docking and neurotransmitter release. *Lamp5* localizes in the synapse, where it may play a regulatory role in GABAergic synaptic transmission [40]. Another hub gene in this network, *Prkcz,* has an important role in hippocampal long term potentiation and learning [42]. Its expression mediates the storage of specific forms of long term memory [38]. The negative association between this network and injury is consistent with the impact that TBI is known to have on memory.

The network representing ME grey60 was associated with “oxidation-reduction process” and “aging”. This module was differentially expressed dependent on *Abca1* copy number; the module was downregulated in *Abca1*^+/−^ mice, and upregulated in *Abca1*^+/+^ mice. The network was built around hub genes involved in the mitochondrial respiratory chain, including a number of the NADH hydrogenase subunits, such as *ND1*, *ND2*, *ND4*, *ND5* and *ND6*, as well as, *COX1*, *Atp5j2*, and *CYTB*. Mitochondrial dysfunction and dysfunctional energy metabolism are early pathological features of multiple neurological diseases, including Alzheimer’s disease, Parkinson’s disease and Huntington’s disease [34, 46]. Perturbations in the mitochondrial respiratory chain results in decreased ATP synthesis, the generation of free radicals and oxidative damage resulting in neuronal dysfunction and apoptosis [30]. HDL and HDL-associated lipids play key roles in the regulation and preservation of mitochondrial function [43]. ABCA1 is an essential mediator of HDL formation, which may explain the negative correlation between *Abca1*^+/−^ mice and this network.

ME turquoise correlated with the groups by injury status, however, the module separated into distinct gene clusters representing unique biological processes. Using the pheatmap function, we were able to separate ME turquoise into 2 sub-modules by hierarchical clustering. The clusters were separated based on injury status and the direction of gene expression. The first cluster was larger and consisted of genes upregulated by injury. This cluster represented the “immune response” and the network was built from several microglia-specific genes including *Trem2, Tyrobp, Hexb,* and *Cd68.* Although there was no specific modulatory effect of *APOE* isoform or *Abca1* copy number on the module, the expression of the module genes was much higher in *E4/Abca1*^*+/*−^ injured mice, which is consistent with our other results*.*

ABCA1 is a major regulator of cholesterol transport and an essential mediator of high density lipoprotein generation [22]. ABCA1 may have a crucial role in the response to TBI by providing essential cholesterol and phospholipids required for repair. However, ABCA1 may also influence the TBI response through its modulatory effects on the inflammatory response. Mice lacking brain ABCA1 exhibit increased neuroinflammation, and in particular have an increased microglial pro-inflammatory response [19]. The effect of ABCA1 on inflammation could also occur through its functional role in mediating cholesterol efflux onto lipid-poor apolipoprotein, including APOE. It was previously shown that the loss of ABCA1 function results in a reduction of APOE, and data from experimental animals show that *Abca1* deficiency abolishes the lipidation of APOE [21]. The isoform-dependent effect of APOE is possibly driven by lipidation status, which has been shown to affect its stability and degradation rate. Our study shows that ABCA1 haploinsufficiency increased expression of the microglia sensome genes in an APOE isoform dependent manner, which suggests gene-gene interactions as a possible mechanism for worsened outcomes after TBI in *APOEε4* carriers.

## Conclusions

Our results suggest a possible role for *Abca1* haplodeficiency on the response to TBI in APOE4 TBI mice at a transcriptional level. When we compared *Abca1*^+/+^ mice to *Abca1*^+/−^ mice by injury status and isoform, we found that the lack of one copy of *Abca1* significantly increased the expression of microglia sensome genes only in *APOE4* TBI mice. This was consistent with the higher expression of the common, upregulated genes, which were associated with immune response. Furthermore, *E4/Abca1*^*+/−*^ showed the highest expression of the immune response gene network, which also included microglia-specific hub genes, *Trem2, Tyrobp, Hexb,* and *Cd68.* Our results suggest that gene-gene interactions can modify the response of *APOE4* mice to harmful effects.

## Abbreviations

ABCA1: ATP Binding Cassette Transporter A1
AD: Alzheimer’s disease
APOE: Apolipoprotein E
CCI: Controlled Cortical Impact
GO: Gene Ontology
ME: Module Eigengene
RNA-seq: RNA-sequencing
TBI: Traumatic brain injury
WGCNA: Weighted Gene Co-expression Network Analysis

## References

[1] Acosta SA, Tajiri N, Shinozuka K, Ishikawa H, Grimmig B, Diamond DM (2013). Long-term upregulation of inflammation and suppression of cell proliferation in the brain of adult rats exposed to traumatic brain injury using the controlled cortical impact model. PLoS One.

[2] Alexander S, Kerr ME, Kim Y, Kamboh MI (2007). Apolipoprotein E4 allele presence and functional outcome after severe traumatic brain injury. J Neurotrauma.

[3] Anthonymuthu TS, Kenny EM, Bayir H (2016). Therapies targeting lipid peroxidation in traumatic brain injury. Brain Res.

[4] Barger SW, Harmon AD (1997). Microglial activation by Alzheimer amyloid precursor protein and modulation by apolipoprotein E. Nature.

[5] Bazarian JJ, Cernak I, Noble-Haeusslein L, Potolicchio S, Temkin N (2009). Long-term neurologic outcomes after traumatic brain injury. J Head Trauma Rehabil.

[6] Blocquel D, Li S, Wei N, Daub H, Sajish M, Erfurth ML (2017). Alternative stable conformation capable of protein misinteraction links tRNA synthetase to peripheral neuropathy. Nucleic Acids Res.

[7] Brooks-Wilson A, Marcil M, Clee SM, Zhang LH, Roomp K, van Dam M (1999). Mutations in ABC1 in tangier disease and familial high-density lipoprotein deficiency. Nature Genet.

[8] Carter AY, Letronne F, Fitz NF, Mounier A, Wolfe CM, Nam KN (2017). Liver X receptor agonist treatment significantly affects phenotype and transcriptome of APOE3 and APOE4 Abca1 haplo-deficient mice. PLoS One.

[9] Castranio EL, Mounier A, Wolfe CM, Nam KN, Fitz NF, Letronne F (2017). Gene co-expression networks identify Trem2 and Tyrobp as major hubs in human APOE expressing mice following traumatic brain injury. Neurobiol Dis.

[10] Centers for Disease C, Prevention (2013). CDC grand rounds: reducing severe traumatic brain injury in the United States. MMWR Morb Mortal Wkly Rep.

[11] Chamelian L, Reis M, Feinstein A (2004). Six-month recovery from mild to moderate traumatic brain injury: the role of APOE-epsilon4 allele. Brain.

[12] Crawford FC, Vanderploeg RD, Freeman MJ, Singh S, Waisman M, Michaels L (2002). APOE genotype influences acquisition and recall following traumatic brain injury. Neurology.

[13] Das M, Mohapatra S, Mohapatra SS (2012). New perspectives on central and peripheral immune responses to acute traumatic brain injury. J Neuroinflammation.

[14] Diaz-Arrastia R, Gong Y, Fair S, Scott KD, Garcia MC, Carlile MC (2003). Increased risk of late posttraumatic seizures associated with inheritance of APOE epsilon4 allele. Arch Neurol.

[15] Draper K, Ponsford J (2008). Cognitive functioning ten years following traumatic brain injury and rehabilitation. Neuropsychology.

[16] Faul M, Coronado V (2015). Epidemiology of traumatic brain injury. Handb Clin Neurol.

[17] Fitz NF, Cronican AA, Saleem M, Fauq AH, Chapman R, Lefterov I (2012). Abca1 deficiency affects Alzheimer's disease-like phenotype in human ApoE4 but not in ApoE3-targeted replacement mice. J Neurosci.

[18] Ghroubi S, Feki I, Chelly H, Elleuch MH (2016). Neuropsychological and behavioral disorders and their correlations with the severity of the traumatic brain injury. Ann Phys Rehabil Med.

[19] Karasinska JM, de Haan W, Franciosi S, Ruddle P, Fan J, Kruit JK (2013). ABCA1 influences neuroinflammation and neuronal death. Neurobiol Dis.

[20] Kim J, Basak JM, Holtzman DM (2009). The role of apolipoprotein E in Alzheimer's disease. Neuron.

[21] Koldamova R, Staufenbiel M, Lefterov I (2005). Lack of ABCA1 considerably decreases brain ApoE level and increases amyloid deposition in APP23 mice. J Biol Chem.

[22] Koldamova R, Fitz NF, Lefterov I (2014) ATP-binding cassette transporter A1: from metabolism to neurodegeneration. Neurobiol Dis 72 Pt A:13–21. 10.1016/j.nbd.2014.05.007 10.1016/j.nbd.2014.05.007 PMC4302328 24844148

[23] Laskowitz DT, Fillit H, Yeung N, Toku K, Vitek MP (2006) Apolipoprotein E-derived peptides reduce CNS inflammation: implications for therapy of neurological disease. Acta Neurol Scand (Suppl 185):15–20. 10.1111/j.1600-0404.2006.00680.x 10.1111/j.1600-0404.2006.00680.x 16866906

[24] Li X, Montine KS, Keene CD, Montine TJ (2015). Different mechanisms of apolipoprotein E isoform-dependent modulation of prostaglandin E2 production and triggering receptor expressed on myeloid cells 2 (TREM2) expression after innate immune activation of microglia. FASEB J.

[25] Liliang PC, Liang CL, Weng HC, Lu K, Wang KW, Chen HJ *et al.* (2010) Tau proteins in serum predict outcome after severe traumatic brain injury. J Surg Res 160:302–307. 10.1016/j.jss.2008.12.022 10.1016/j.jss.2008.12.022 19345376

[26] Lynch JR, Morgan D, Mance J, Matthew WD, Laskowitz DT (2001). Apolipoprotein E modulates glial activation and the endogenous central nervous system inflammatory response. J Neuroimmunol.

[27] Maas AI, Stocchetti N, Bullock R (2008). Moderate and severe traumatic brain injury in adults. Lancet Neurol.

[28] Mannix RC, Zhang J, Park J, Zhang X, Bilal K, Walker K (2011). Age-dependent effect of apolipoprotein E4 on functional outcome after controlled cortical impact in mice. J Cereb Blood Flow Metab.

[29] Morganti JM, Jopson TD, Liu S, Riparip L-K, Guandique CK, Gupta N (2015). CCR2 antagonism alters brain macrophage polarization and ameliorates cognitive dysfunction induced by traumatic brain injury. J Neurosci.

[30] Muller WE, Eckert A, Kurz C, Eckert GP, Leuner K (2010). Mitochondrial dysfunction: common final pathway in brain aging and Alzheimer's disease--therapeutic aspects. Mol Neurobiol.

[31] Nam KN, Mounier A, Fitz NF, Wolfe C, Schug J, Lefterov I (2016). RXR controlled regulatory networks identified in mouse brain counteract deleterious effects of Abeta oligomers. Sci Rep.

[32] Nam KN, Mounier A, Wolfe CM, Fitz NF, Carter AY, Castranio EL (2017). Effect of high fat diet on phenotype, brain transcriptome and lipidome in Alzheimer's model mice. Sci Rep.

[33] Nam KN, Wolfe CM, Fitz NF, Letronne F, Castranio EL, Mounier A (2018). Integrated approach reveals diet, APOE genotype and sex affect immune response in APP mice. Biochim Biophys Acta.

[34] Pathania D, Millard M, Neamati N (2009). Opportunities in discovery and delivery of anticancer drugs targeting mitochondria and cancer cell metabolism. Adv Drug Deliv Rev.

[35] Perez-Garcia G, Gama Sosa MA, De Gasperi R, Lashof-Sullivan M, Maudlin-Jeronimo E, Stone JR et al (2016) Chronic post-traumatic stress disorder-related traits in a rat model of low-level blast exposure. Behav brain res. In: 10.1016/j.bbr.2016.09.061 10.1016/j.bbr.2016.09.061 PMC11181290 27693852

[36] Plassman BL, Grafman J (2015). Traumatic brain injury and late-life dementia. Handb Clin Neurol.

[37] Ponsford J, Rudzki D, Bailey K, Ng KT (2007). Impact of apolipoprotein gene on cognitive impairment and recovery after traumatic brain injury. Neurology.

[38] Shema R, Sacktor TC, Dudai Y (2007). Rapid erasure of long-term memory associations in the cortex by an inhibitor of PKM zeta. Science.

[39] Simon DW, McGeachy MJ, Bayir H, Clark RS, Loane DJ, Kochanek PM (2017). The far-reaching scope of neuroinflammation after traumatic brain injury. Nat Rev Neurol.

[40] Tiveron MC, Beurrier C, Ceni C, Andriambao N, Combes A, Koehl M *et al.* (2016) LAMP5 fine-tunes GABAergic synaptic transmission in defined circuits of the mouse brain. PLoS One 11:e0157052. 10.1371/journal.pone.0157052 10.1371/journal.pone.0157052 PMC4896627 27272053

[41] Trushina E, Dutta T, Persson XM, Mielke MM, Petersen RC (2013). Identification of altered metabolic pathways in plasma and CSF in mild cognitive impairment and Alzheimer's disease using metabolomics. PLoS One.

[42] Wang S, Sheng T, Ren S, Tian T, Lu W (2016). Distinct roles of PKCiota/lambda and PKMzeta in the initiation and maintenance of hippocampal long-term potentiation and memory. Cell Rep.

[43] White CR, Datta G, Giordano S (2017). High-density lipoprotein regulation of mitochondrial function. Adv Exp Med Biol.

[44] Whitnall L, McMillan TM, Murray GD, Teasdale GM (2006). Disability in young people and adults after head injury: 5-7 year follow up of a prospective cohort study. J Neurol Neurosurg Psychiatry.

[45] Yang SH, Gangidine M, Pritts TA, Goodman MD, Lentsch AB (2013) Interleukin 6 mediates neuroinflammation and motor coordination deficits after mild traumatic brain injury and brief hypoxia in mice. Shock (Augusta, Ga) 40:471–475 10.1097/SHK.0000000000000037 PMC4218737 24088994

[46] Yu H, Lin X, Wang D, Zhang Z, Guo Y, Ren X (2018). Mitochondrial molecular abnormalities revealed by proteomic analysis of hippocampal organelles of mice triple transgenic for Alzheimer disease. Front Mol Neurosci.

[47] Zhang L, Xing G, Tie Y, Tang Y, Tian C, Li L (2005). Role for the pleckstrin homology domain-containing protein CKIP-1 in AP-1 regulation and apoptosis. EMBO J.

[48] Zhao W, Langfelder P, Fuller T, Dong J, Li A, Hovarth S (2010). Weighted gene coexpression network analysis: state of the art. J Biopharm Stat.

